# Supplementary material for: Preneoplastic cells switch to Warburg metabolism from their inception exposing multiple vulnerabilities for targeted elimination
Source: Oncogenesis. 2024 Jan 25;13(1):7. doi: 10.1038/s41389-024-00507-4 (PMC10810875; doi:10.1038/s41389-024-00507-4)
Supplement: Supplementary file 1 — Supplemental figure legends [file 41389_2024_507_MOESM1_ESM.docx]

**Supplemental Material**

**Supplemental figure 1**

A. Trace annotation for Oxygen Consumption Rate measurement from Seahorse XF® Analyser, for data showed in main figure 2 A, B.

**Supplemental figure 2**

A. Graph shows quantification for anti-active-caspase 3 staining in CAAX cells from larvae treated with mDIVI, metformin, Glucose, showing CAAX cells are not affected by the treatments. (One-way ANOVA analysis with Dunnet’s multiple comparison, n≥9) B. Graph shows quantification for EdU positive CAAX cells in glucose treated larvae compared with untreated control and CAAX cells are not affected. Mann-Whitney test, n≥14, p>0.99. C Graph shows quantification for EdU positive CAAX cells in mdivi treated larvae compared with untreated control and CAAX cells are not affected. Mann-Whitney test, n≥14, p=0.1933. D. Graph shows quantification for EdU positive CAAX cells in metformin treated larvae compared with untreated control and CAAX cells are not affected. Unpaired *t* test, n≥11, p=0.3957. E. Graph shows quantification for EdU positive CAAX cells in lonidamine treated larvae compared with untreated control and CAAX cells are not affected. Unpaired *t* test, n≥12, p=0.1169.
