# Supplementary figures and images for: Preneoplastic cells switch to Warburg metabolism from their inception exposing multiple vulnerabilities for targeted elimination
Source: Oncogenesis. 2024 Jan 25;13(1):7. doi: 10.1038/s41389-024-00507-4 (PMC10810875; doi:10.1038/s41389-024-00507-4)

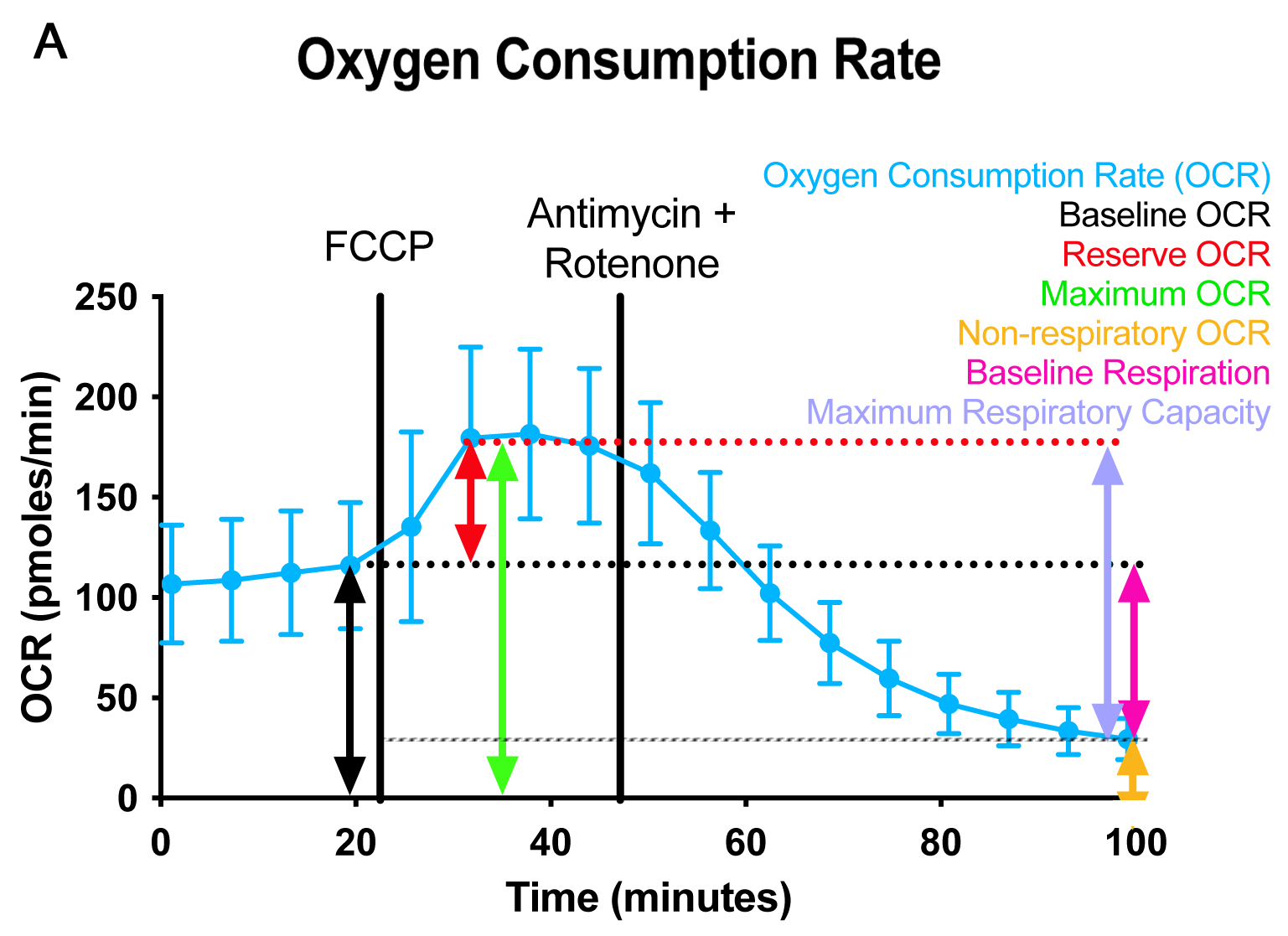

Supplement: Supplementary file 2 — Supplemental figure 1 [file 41389_2024_507_MOESM2_ESM.tif]

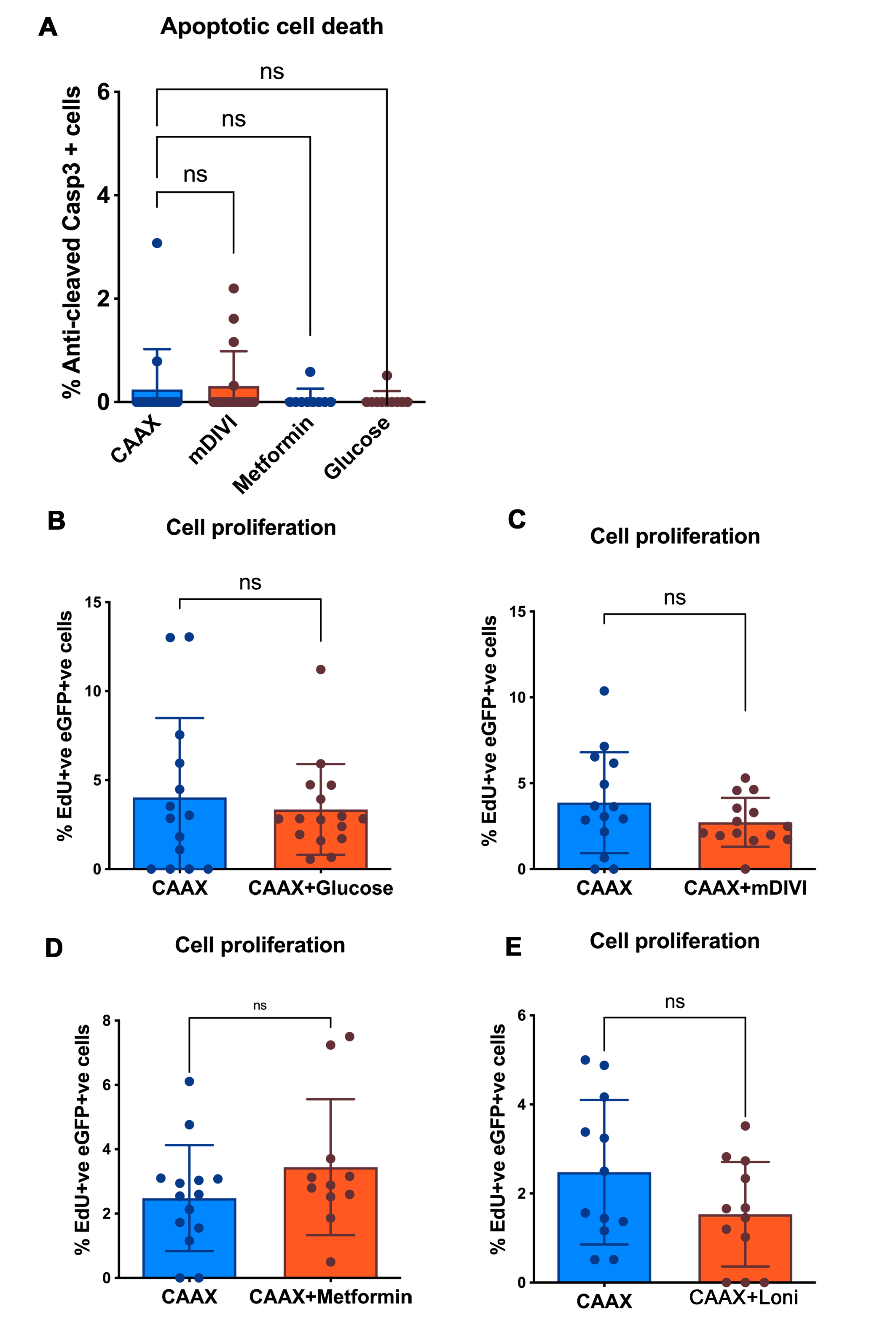

Supplement: Supplementary file 3 — Supplemental figure 2 [file 41389_2024_507_MOESM3_ESM.tif]
